# Function and mechanism of tumor suppressor gene LRRC4/NGL-2

**DOI:** 10.1186/1476-4598-13-266

**Published:** 2014-12-19

**Authors:** Peiyao Li, Gang Xu, Guiyuan Li, Minghua Wu

**Affiliations:** Hunan Cancer Hospital and the Affiliated Tumor Hospital of Xiangya Medical School Central South University, Changsha, Hunan China; Cancer Research Institute, Central South University, Changsha, Hunan 410078 P.R. China; Key Laboratory of Carcinogenesis and Cancer Invasion, Ministry of Education, Shanghai, China; Key Laboratory of Carcinogenesis, Ministry of Health, Changsha, Hunan China; Medical College, University of South China, Hengyang, Hunan China

**Keywords:** Leucine-rich repeat, Netrin, Netrin-G ligand, miRNA, Methylation, Glioma

## Abstract

LRRC4/NGL-2 (Leucine rich repeat containing 4/Netrin-G ligand-2), a relatively specific expressed gene in brain tissue, is a member of the LRRC4/ NGL (netrin-G ligand) family and belongs to the superfamily of LRR proteins. LRRC4/NGL-2 regulates neurite outgrowth and lamina-specific dendritic segmentation, suggesting that LRRC4/NGL-2 is important for the development of the nervous system. In addition, LRRC4/NGL-2 has been identified as a tumor suppressor gene. The overexpression of LRRC4/NGL-2 suppresses glioma cell growth, angiogenesis and invasion through complicated signaling regulation networks. LRRC4/NGL-2 also has the ability to form multiphase loops with miRNA, transcription factors and gene methylation modification; the loss of LRRC4/NGL-2 function may be an important event in multiple biological processes in gliomas. In summary, LRRC4/NGL-2 is a critical gene in the normal development and tumorigenesis of the nervous system.

## Introduction

The leucine-rich repeat (LRR) superfamily is composed of a notably heterogeneous group of proteins containing leucine-rich domains (LRR domain) known to mediate highly specific protein-protein interaction or cell adhesion. Many of LRR-containing proteins are involved in the differentiation and development of normal nervous tissues [1–6].

The LRRC4 (GenBank accession No. AF196976) gene was cloned and characterized from human chromosome 7q31-32 using a computer-assisted positional cloning strategy combining 5'-RACE [7]. Lin et al. (2003) determined that there is a similarity of phenotype and sequence between LRRC4 and the netrin-G1 ligand [8]. Afterwards, Kim et al. demonstrated that LRRC4 directly interacts with netrin-G2, indicating that it is a ligand for netrin-G2; thus, LRRC4 is also named NGL-2 (netrin-G ligand-2) [9].

A genomic database analysis has since identified that LRRC4 is a member of the LRRC4 (NGL, netrin-G ligand) family and belongs to the superfamily of LRR proteins. Moreover, there are three known members in the LRRC4 family; LRRC4C (NGL-1), LRRC4 (NGL-2) and LRRC4B (NGL-3) [10]. LRRC4/NGL-2 displays down-regulation or expression deletion in primary brain tumor biopsies and has the potential to suppress brain tumor growth [7].

## Structure and distribution of LRRC4/NGL-2

**Figure 1:**
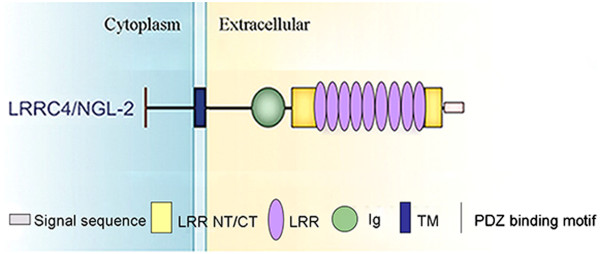
**Domain structure of LRRC4/NGL-2.** LRR NT/CT, N- or C-terminal LRR; LRR, leucine-rich repeat; Ig, immunoglobulin; TM, transmembrane; PDZ, PSD-95/Dlg/ZO-1.

LRRC4/NGL-2 is a member of the LRR superfamily. It contains two segments at its N-terminus and C-terminus for sequences representing a putative signal peptide and a transmembrane region, respectively. Following the signal peptide, the core LRR region consists of nine LRRs accompanied by typical amino-flanking (AF) and carboxy-flanking (CF) clusters [1]. The amino-terminal LRR-flanking domain contains cysteines in a C–X3–C–X–C–X8–C pattern, whereas the carboxy–terminal LRR-flanking domain conforms to a P–X2–C–X–C–X19–C–X2–C pattern. Cytosine clusters are known to enhance the stability of the central LRRs [11]. Adjacent to the CF region, one IgC2 domain is identified [8]. The transmembrane domain is followed by a cytoplasmic region, which ends with a PDZ domain-binding motif [12]. LRRC4C/NGL-1, LRRC4/NGL-2, and LRRC4B/NGL-3 share the same domain structure (Figure 1). The LRRC4 family displays a sequence identity of approximately 57-61% in the LRR and Ig domains, but their cytoplasmic domains show essentially no sequence identity, except in the PDZ-binding motif. Moreover, the extracellular region immediately preceding the transmembrane domain varies significantly among LRRC4s, suggesting that each LRRC4 may have a distinct function [10].

LRRC4/NGL-2 displayed a brain-specific expression pattern both in humans and mice. LRRC4/NGL-2 was detected in the human brain but not in other normal tissues including the heart, lung, liver and so on [13, 14]. The mRNAs for LRRC4C/NGL-1 and LRRC4B/NGL-3 are mainly expressed in the brain, with minor expression in the liver and heart, respectively. The transcripts of the LRRC4 family members are distributed in distinct regions of embryonic and postnatal brains [9, 8, 13, 15]. LRRC4/NGL-2 expression in both the human and mice brain was limited to the following areas: cerebellum, cerebral cortex, occipital pole, frontal lobe, temporal lobe and putamen [13]. The LRRC4C/NGL-1 and LRRC4B/NGL-3 proteins have been indicated to be mainly expressed in the brain but not in other tissues [9]. The absence of LRRC4C/NGL-1 and LRRC4B/NGL-3 protein expression in the liver and heart, respectively, is dissimilar to their mRNA expression patterns. The LRRC4C/NGL-1 protein is detected in the hippocampus, neocortex, and piriform cortex (Table 1). Within neurons, LRRC4/NGL-2 and LRRC4C/NGL-1 are selectively localized to distinct dendritic segments [15]. LRRC4/NGL family members are mainly localized to the postsynaptic side of excitatory synapses, and NGL-1 interacts with netrin-G1, NGL-2 with netrin-G2, and NGL-3 with LAR, which play important roles in the development of axons, dendrites and synapses [10].

**Table 1:** **The expression distribution of the LRRC4 family in brain tissues**

LRRC4 family	Protein distribution
LRRC4/NGL-2	cerebellum, cerebral cortex, occipital pole, frontal lobe, temporal lobe, and putamen
LRRC4C/NGL-1	hippocampus, neocortex, and piriform cortex
LRRC4B/NGL-3	similar brain-specific

## Role of LRRC4/NGL-2 in maintaining normal function in the central nervous system

A number of leucine-rich repeat proteins with LRRs and Ig regions, which seem to serve similar functions, have been found to be expressed predominantly in cells of nervous tissues [8, 16, 17], and are involved in the differentiation and development of normal nervous tissues [1, 2]. LRRC4C/NGL-1, LRRC4/NGL-2 and LRRC4B/NGL-3 share the same domain structure. The structure character of LRRC4s implies that these genes might be involved in the development and differentiation of the nervous system. LRRC4C/NGL-1 was first identified as a ligand for netrin-G1 [8]. LRRC4C/ NGL-1 has been found to interact with netrin-G1 but not with netrin-G2. In addition, LRRC4/NGL-2 interacts with netrin-G2 but not with netrin-G1 [9]. LRRC4B/NGL-3 does not interact with either netrin-G1 or netrin-G2 but was determined to interact with the receptor tyrosine phosphatase LAR [10, 18]. The interaction between netrin-G1 and LRRC4C/NGL-1 has been implicated in the regulation of axonal outgrowth and migration [8]. The LRRC4C/NGL1-netrinG1 and LRRC4/NGL2-netrinG2 interactions have been implicated in the lamina-specific segmentation of dendrites [15]. In the early development of the brain, the expression of mLRRC4/NGL-2 increased with age, suggesting a possible role of LRRC4/NGL-2 in brain development [13]. LRRC4/NGL-2 regulates the formation of excitatory synapses through the recruitment of pre- and postsynaptic proteins [19], participates in the differentiation of neuron and glia cells, and promotes neurite outgrowth [20]. In addition,LRRC4/NGL-2 formed a complex with the NMDA receptor subunits involved in the regulation of NMDA(N-methyl-D-aspartate) receptor-mediated excitatory synaptic transmission [9]. LRRC4/NGL-2 is localized to the dendritic segment in the stratum radiatum of CA1(Cornu Ammonis 1 area) and functions as a key regulator of input-specific synapse development, which is critical for the functional integration of distinct inputs [21]. LRRC4/NGL-2 was found to localize selectively to the tips of HC(horizontal cell) axons, which form reciprocal connections with rods; moreover, LRRC4/NGL-2 works as a central component of pathway-specific development in the outer retina [22]. LRRC4B/NGL-3–LAR interaction is capable of mediating cell aggregation [23]. LRRC4B/NGL-3 might be involved in the maturation of excitatory synapses. A suggested function of the LRRC4B/NGL-3–LAR interaction is synapse formation [23]. The results from studies on the LRRC4 family suggest that LRRC4s play important roles in maintaining normal function in the central nervous system. In addition to the functions in neuronal cell types, LRRC4/NGL proteins play a role in glial cells [10].

## Aberrant expression of LRRC4/NGL-2 in tumors of the central nervous system

LRRC4/NGL-2 was found to be predominantly expressed in the normal brain tissues, though it was deleted or down-regulated in primary brain tumor biopsies (up to 87.5% in gliomas, 80.9% in meningiomas and 85.2% in pituitary and other brain tumors) [7]. LRRC4/NGL-2 was highly specific in brain tissue and grade I gliomas (WHO), but it was reduced or absent in grade II-III gliomas and absent in glioblastoma (WHO, grade IV) [24]. Thus, the loss of LRRC4/NGL-2 function may directly contribute to the increasing tumor grade and is a late event in the pathogenesis of gliomas. Furthermore, LRRC4/NGL-2 is downregulated expression in pituitary adenoma, polymorphisms or haplotypes in the LRRC4/NGL-2 and may have important research significance by predicting the risk of pituitary adenoma [25].

## Role of LRRC4/NGL-2 as a tumor suppressor gene in glioma tumorigenesis

LRRC4/NGL-2 significantly inhibited glioma cell proliferation and greatly reduced its capacity to form colonies [24]. Furthermore, it reduced the growth and malignant grade of xenografts arising from glioma cells. The ultrastructure of glioma cell Tet-on-LRRC4/NGL-2 underwent a significant change, the nucleo-cytoplasmic ratio lessened, the nuclear shape became regular, heterochromatin in nuclei decreased while euchromatin increased, the volume of nucleoli lessened, rough ER increased significantly, the Golgi apparatus was well-developed and Golgi vesicles increased and were regularly arranged, most mitochondria were oval and their crista grew in number and were regularly arranged, and finally polyribosomes reduced while free ribosomes increased [26]. In addition, it has the ability to inhibit the invasion and angiogenesis of glioma cells.

LRRC4/NGL-2 suppressed glioma cell proliferation by delaying the cell cycle in late G1 and did not induce apoptosis of tumor cells [26]. Furthermore, LRRC4/NGL-2 inhibited the glioma tumor cell invasion through regulating the expression of the invasion-related molecules including CD44, MMP16, TB10 and annexin A2 [27]. In addition, LRRC4/NGL-2 might be a negative regulator of the RPTP-zeta receptor, contributing to the suppression of the invasion ability of glioma cells [28]. The reintroduction of LRRC4/NGL-2 might inhibit the expression of CXCR4 and SDF-1α/CXCR4 axis-mediated cell invasion in vitro [29]. Moreover, LRRC4/NGL-2 inhibits glioblastoma cell proliferation, migration and angiogenesis by downregulating pleiotropic cytokine expression and response [30]. Together, these findings demonstrated that LRRC4/NGL-2 can be identified as a tumor suppressor gene in the tumorigenesis of glioma. The inhibitory effect of LRRC4/NGL-2 on cell proliferation and invasion is dependent on its LRR cassette domain but not on IgC2 or Tm domain. In the LRR cassette domain, the third LRR motif of the core LRR is found to be indispensable for the function of LRRC4/NGL-2, and it plays a crucial role as a “proliferation-inhibition switch”. The seventh LRR motif plays a crucial role as an “invasion-inhibition switch” [24].

## Signaling transduction network of LRRC4/NGL-2 in glioma

**Figure 2:**
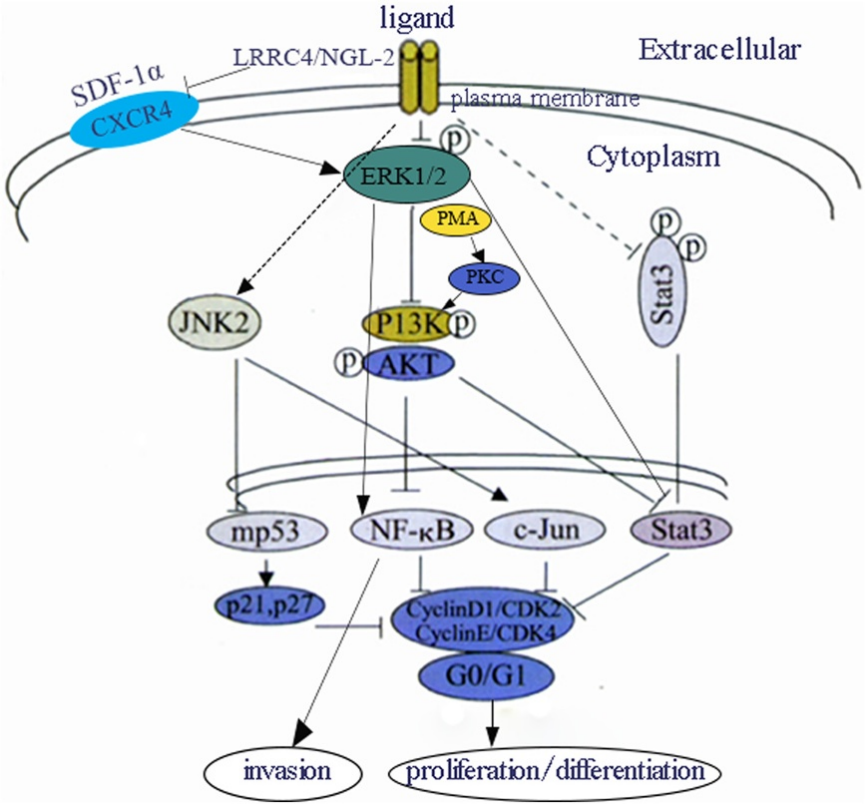
**Schematic** ***diagram*** **showing the signaling regulation network of LRRC4/NGL-2 in glioma.** LRRC4/NGL-2 can delay the cell cycle in late G1 by increasing the expression of cell cycle inhibitory molecules (p21, p27) and reducing the expression of cell cycle regulatory proteins (CyclinD1, CDK2, CyclinE, CDK4) via the inhibition of K-Ras/c-Raf/ERK/MAPK, PI-3K/AKT/NF- κB, p70S6/PKC and STAT3, and the upregulation of the JNK2/c-Jun/mp53 signaling pathway. LRRC4 /NGL-2 inhibits the expression of CXCR4 and SDF-1 alpha/CXCR4 axis-mediated downstream intracellular pathways leading to proliferate, chemotactic and invasive effects.

To further analyze the mechanisms of LRRC4/NGL-2 on the suppression of tumorigenesis and progression in gliomas, Zhang et al. investigated alterations in gene expression related to the neurobiology of the Atlas array and found that 1) The overexpression of LRRC4/NGL-2 can elevate the expression levels of certain cell cycle progression regulators, such as RAP1GAP, ephrin-B3, somatostatin receptors, PTPN and NT-3, and regulate suppressing cell cycle progression by impacting on Raf/Rap/Ras pathways; 2) the overexpression of LRRC4/NGL-2 can down-regulate the expression of genes involved in tumor invasion and metastasis, including CD44, MMP16, Thymosin beta-10 and AnnexinA2; and 3) LRRC4/NGL-2 may act as a receptor for adhesion molecule Netrin-G2 by participating in the inhibition of tumorigenesis and suppression of glioma tumorigenesis by preventing the synthesis and release of toxic neurotransmitters [27]. LRRC4/NGL-2 can delay the cell cycle in late G1 by increasing the expression of cell cycle inhibitory molecules (p21, p27) and reducing the expression of cell cycle regulatory proteins (CyclinD1, CDK2, CyclinE, CDK4) via the down-regulation of growth factors (IGF, bFGF, EGF, PDGF, VEGF SDF-1α) or receptors, the inhibition of K-Ras/c-Raf/ERK/MAPK, PI-3 K/AKT/NF- κB, p70S6/PKC and STAT3, and the upregulation of the JNK2/c-Jun/mp53 (mutant p53) signaling pathway [24, 27]. Moreover, the PMA-stimulated activation of PKC activates PI3K/Akt, but not pERK, in the presence of LRRC4/NGL-2, which is consistent with the fact that PKC is located downstream of ERK but upstream of PI3K/Akt [24]. The reintroduction of LRRC4/NGL-2 in U251 cells inhibits the expression of CXCR4 (CXC chemokine receptor 4) and SDF-1 (stromal cell-derived factor-1) alpha/CXCR4 axis-mediated downstream intracellular pathways such as ERK1/2 and Akt leading to proliferate, chemotactic and invasive effects (Figure 2) [29].

## Epigenetic changes of LRRC4/NGL-2 in glioma

LRRC4/NGL-2 was a candidate tumor suppressor gene that may be involved in the pathogenesis of malignant glioma. However, no genetic alterations of the LRRC4/NGL-2 coding region were found in glioma [24]. The methylation-mediated inactivation of LRRC4/NGL-2 was found to be a frequent and glioma-specific event. Furthermore, the methylation of LRRC4/NGL-2 was detected in both the early and late stages of glioma, indicating that the inactivation of the LRRC4/NGL-2 gene might be essential in the early development of glioma and persist through the course of development [31]. Both DNA methylation and histone modification are not isolated epigenetic events; there are cross-talks between them. Trimethyl-H3 (Lys9) is correlated with LRRC4/NGL-2 promoter methylation, which suppresses LRRC4/NGL-2 expression and H3 acetylation, while trimethyl-H3 (lys4) is correlated with LRRC4/NGL-2 promoter demethylation, which activates LRRC4/NGL-2 expression [32].

The other dysregulation mechanisms of LRRC4/NGL-2 in glioma result from the miRNA-dependent regulatory network.

## LRRC4/NGL-2 as the core of miRNA-dependent multiphase loops in glioma

*MicroRNAs (miRNAs) are approximately 22 nt non-coding RNAs that interfere with the translation of coding mRNAs in a sequence-specific manner*[33]*.* MiRNAs function as tumor suppressors or oncogenes to induce cellular transformation and tumorigenesis [34–36]. *MiRNA-mediated gene regulation is now considered to be important in numerous biological processes and is also recognized as being crucial for regulating gene expression in tumors*[37, 38]*.* Several key studies on miRNA expression patterns in gliomas have been completed to date. The abnormal expression of some miRNAs (miR-12b, miR-21, miR-221, miR-128, miR-181a, miR-181b and miR-181c et al.) were found in gliomas and might be involved in tumorigenesis and development [25, 39–41]. A greater number of studies have been completed via the siRNA profiling of glioma; correlations between siRNA templates, their target genes, and candidate miRNA may help experimentally identify candidate miRNA targets in glioma [42]. *LRRC4/NGL-2* is a target gene of some miRNAs (such as miR-182 and miR-381); meanwhile, it is capable of regulating miRNAs as a tumor suppressor and forms multiphase loops with miRNAs, transcription factors and gene methylation modification in glioma [43–46].

### The LRRC4*/NGL-2*-AP-2-miR-182- LRRC4*/NGL-2*Loop

*There is a set of cis-acting elements in the promoters of many miRNAs, which are regulated by a series of upstream transcription factors (TFs)*[47]*. Similar to the transcription of the protein-coding gene, TFs regulate miRNA expression at the transcriptional level. Further, miRNA might influence the expression of its target genes and constitute a TF-miRNA-gene regulatory network*[48, 49]*.* Genes not only modulate the expression of miRNA target genes by influencing TF-mediated miRNAs expression but also regulate TFs through miRNAs [50, 51]. In the regulatory processes where target genes are modulated by both miRNAs and transcription factors, TFs regulate miRNAs, and miRNAs suppress TFs. In this way, multiphase loops might be constituted at last [52–54].

*LRRC4/NGL-2* is a target gene of miR-381 and miR182; meanwhile, the overexpression of *LRRC4/NGL-2* downregulated the expression of miR-381 and miR182 in glioma cells [43–45]. The interactions between miR-381/miR182 and *LRRC4/NGL-2* were identified to be involved in the pathological progression of astrocytoma [43]. BRD7, a transcriptional cofactor for p53, is highly expressed and negatively correlated with *LRRC4/NGL-2* expression in gliomas. Disturbing miR-182 and miR-381 inhibits the expression of BRD7, arrests glioma cells in the G0/G1 phase of the cell cycle, inhibits glioma cell growth and induces differentiation of glioma cells to astrocyte-like cell by upregulating *LRRC4/NGL-2* and suppressing the *LRRC4/NGL-2*-mediated binding of AP-2/SP1/E2F6/c- Myc to BRD7 in ERK/MAPK and PI-3 K/AKT signal pathways [43, 45].

The transcription of miR-182 was induced by transcription factor AP-2; meanwhile,miR-182 can inhibit the expression of LRRC4*/NGL-2*, and LRRC4*/NGL-2* might inhibit the expression of AP-2 though negatively regulating the ERK/MAPK and PI-3 K/AKT signaling pathways. It was demonstrated that the LRRC4-AP-2-miR-182-LRRC4 loop formed among LRRC4*/NGL-2*, miR-182 and AP-2 was involved in glioma development [43, 45, 46].

### The LRRC4*/NGL-2*-miR-185/SP1-DNMT1-LRRC4*/NGL-2*Loop

MiRNAs regulate the epigenetic modification of genes through modulating the expression of DNMT (DNA methyltransferase), maintaining DNA methylation and mediating histone modifications [54–57]. During tumorigenesis, miRNAs not only mediate the methylation of DNAs by regulating DNMT but also can be methylated in its promoter. Thus, a miRNA-DNMT regulatory loop might be formed between miRNAs and DNMT [58–60].

The overexpression of LRRC4/NGL-2 could increase the expression of miR-185, while miR-185 could regulate global methylation by inhibiting DNA methyltransferase DNMT1 and increasing the expression of such hypermethylation genes such as LRRC4/NGL-2 in the end. It was indicated that the LRRC4/NGL-2-miR-185-DNMT1-LRRC4/NGL-2 loop among LRRC4/NGL-2, miR185 and DNMT1 participated in glioma development. In addition, DNMT1 was positively regulated by SP1, and it could increase the expression of LRRC4/NGL-2, while LRRC4/NGL-2 could also inhibit SP1 by negatively regulating the ERK/MAPK and PI-3 K/AKT signal pathway. Therefore, the LRRC4/NGL-2-SP1-DNMT1-LRRC4/NGL-2 loop formed among LRRC4/NGL-2, SP1 and DNMT1 took part in the glioma formation [44, 46]. In addition, CDC42 and RhoA were the direct targets of miR-185. Furthermore, CDC42 and RhoA were inversely correlated with miR-185 expression in gliomas. LRRC4/NGL-2 mediated its tumor suppressor by regulating miR-185 targets CDC42 and RhoA. LRRC4/NGL-2 overexpression inhibited glioma cell growth and invasion through miR-185-mediated CDC42 and RhoA direct regulation and VEGFA indirect regulation [44].

**Figure 3:**
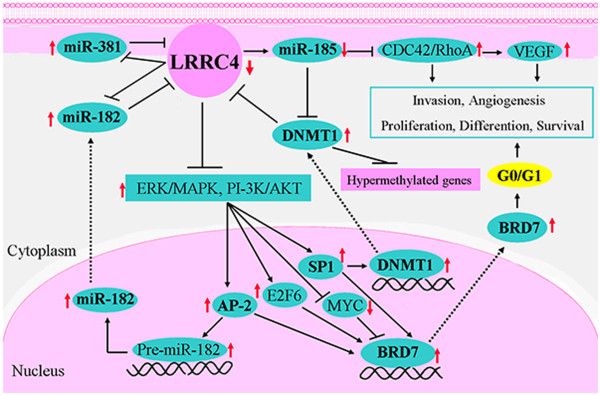
**Schematic** ***diagram*** **showing the multi-regulation loops of LRRC4/NGL-2, miRNAs, TFs and target genes in glioma.** LRRC4/NGL-2-AP-2-miR-182- LRRC4/NGL-2 loop: LRRC4/NGL-2 is a target of miR182; meanwhile, the overexpression of LRRC4/NGL-2 downregulated the expression of miR182. The transcription of miR-182 was induced by AP-2; meanwhile, miR-182 can inhibit the expression of LRRC4/NGL-2, and LRRC4/NGL-2 can inhibit the expression of AP-2 though negatively regulating the ERK/MAPK and PI-3K/AKT signaling pathways. The LRRC4/NGL-2-AP-2-miR-182- LRRC4/NGL-2 loop was formed among LRRC4/NGL-2, miR-182 and AP-2. LRRC4/NGL-2-miR-185-DNMT1-LRRC4/NGL-2 loop: The overexpression of LRRC4/NGL-2 could increase the expression of miR-185, while miR-185 could regulate global methylation by targeting DNMT1 and increasing the expression of LRRC4/NGL-2. LRRC4/NGL-2-SP1-DNMT1-LRRC4/NGL-2 loop: DNMT1 was positively regulated by SP1, and it could increase the expression of LRRC4/NGL-2, while LRRC4/NGL-2 could also inhibit SP1 by negatively regulating the ERK/MAPK and PI-3K/AKT signal pathway.

Briefly, at the time of LRRC4/NGL-2 regulating miRNAs as a tumor suppressor, those miRNAs were found to regulate the binding of transcription factors to DNA in their target-mediated signal pathways by directly targeting genes (such as LRRC4/NGL-2) or regulating the methylation and expression of such hypermethylation genes such as LRRC4/NGL-2 by directly targeting DNA methyltransferase and controlling global methylation. Thus, multiphase loops, which had a core of LRRC4/NGL-2, were formed (Figure 3) [46]. They were LRRC4*/NGL-2*-AP-2-miR-182-LRRC4*/NGL-2*, LRRC4*/NGL-2*-miR-185-DNMT1-LRRC4*/NGL-2* and LRRC4*/NGL-2*-SP1-DNMT1-LRRC4*/NGL-2*. These loops were involved in glioma development with multiple positive feedback formations among them.

## Conclusions

LRRC4/NGL-2, a member of the LRR superfamily thought to be involved in tumorigenesis and the development of the nervous tissues, has the potential to suppress gliomas cell growth, angiogenesis and invasion through complex regulation networks. In addition, LRRC4/NGL-2 has the ability to form multiphase loops with miRNA, transcription factors and target genes. These loops are involved in glioma development with multiple positive feedback formations among them. The results from studies on LRRC4/NGL-2 suggest that LRRC4/NGL-2 is not only a brain-specific gene, but it has been identified as a tumor suppressor gene for gliomas. Therefore, the loss of LRRC4/NGL-2 function may be an important event in multiple biological processes related to gliomas.

There are still many interesting questions that need to be addressed in future studies. Does the missing LRRC4/NGL-2 gene affect the development of the central nervous system? Does the lack of LRRC4/NGL-2 expression result in impaired learning and memory performance? To answer these questions, the role of LRRC4/NGL-2 in central nervous development and function should be verified through in vivo studies employing neuron-specific knockout mice.

## Abbreviations

LRR: Leucine-rich repeat
LRRC4: Leucine rich repeat containing 4
NGL: Netrin-G ligand
AF: Amino-flanking
CF: Carboxy-flanking
Ig: Immunoglobulin
PDZ: PSD-95/Dlg/ZO-1
CXCR4: CXC chemokine receptor 4
SDF-1: Stromal cell-derived factor-1
TF: *transcription factors*
HC: Horizontal cell
CA1: Cornu Ammonis 1 area
NMDA: N-methyl-D-aspartate.
